# An Unsymmetrical Tetrathiafulvalene with a Fused 1,2,5-Thiadiazole Ring and Methylthio Groups

**DOI:** 10.3390/molecules14104266

**Published:** 2009-10-23

**Authors:** Masaaki Tomura, Yoshiro Yamashita

**Affiliations:** 1 Institute for Molecular Science, Myodaiji, Okazaki 444-8585, Japan; 2 Department of Electronic Chemistry, Interdisciplinary Graduate School of Science and Engineering, Tokyo Institute of Technology, Nagatsuta, Midori-ku, Yokohama 226-8502, Japan; E-Mail: yoshiro@echem.titech.ac.jp (Y.Y.)

**Keywords:** tetrathiafulvalene, 1,2,5-thiadiazole, intermolecular S···S heteroatom interaction, one-dimensional molecular tape structure

## Abstract

The title compound, 5-(4,5-dimethylthio-1,3-dithiol-2-ylidene)-1,3-diaza-2,4,6-trithiapentalene (4,5-dimethylthio[1,2,5]thiadiazolotetrathiafulvalene, molecular formula C_8_H_6_N_2_S_7_) crystallizes in the *P*2_1_/*n* space group with one molecule in the asymmetric unit. The molecular framework is planar within 0.19 Å. The molecules form a head-to-tail type of *π*-stacking dimer with an interplanar distance is 3.50(1) Å, where methylthio groups face away from each other. The molecules are also linked by weak intermolecular S···S heteroatom interactions [3.497(1) and 3.572(1) Å] to construct a unique one-dimensional molecular tape structure.

## Introduction

**Figure 1:**
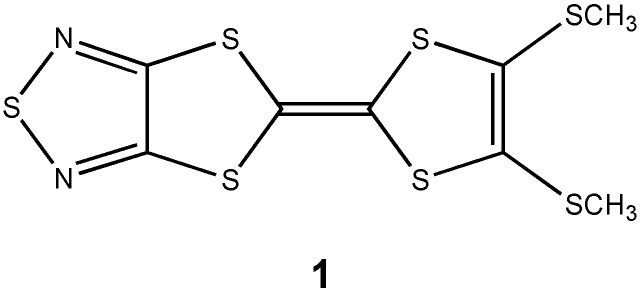
Structure of compound **1**.

Tetrathiafulvalene (TTF) derivatives with a fused 1,2,5-thiadiazole ring have received much attention as component molecules for conducting organic solids [1,2,3,4,5,6,7]. Bis-fused TTF derivatives with a fused 1,2,5-thiadiazole ring have also been developed [8]. Intermolecular interactions caused by S···N and S···S heteroatom contacts may increase the dimensionality in solid states and suppress metal-insulator transitions [9,10]. In addition, such interactions may lead to the formation of unique molecular networks which have special functions such as inclusion properties [11]. We report here the molecular and crystal structure of an unsymmetrical TTF derivative, (**1**, Figure 1), which contains a fused 1,2,5-thiadiazole ring and two methylthio groups. We have found that the crystal structure of **1** differs dramatically from that of an unsymmetrical TTF derivative with a fused 1,2,5-thiadiazole ring and an ethylenedioxy group [6]. The molecules of **1** form a unique one-dimensional molecular tape structure linked by intermolecular S···S heteroatom interactions in the crystalline state.

## Results and Discussion

**Figure 2:**
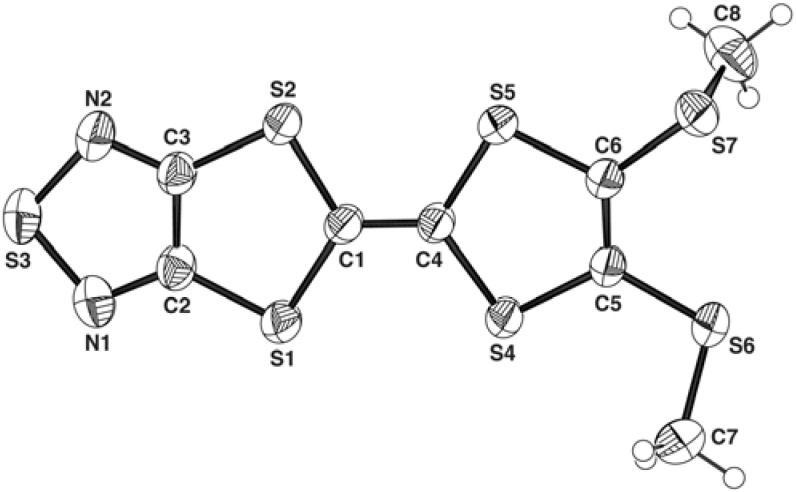
The molecular structure of **1**, with atom labels and 50% probability displacement ellipsoids for non-H atoms and H atoms are shown as small spheres of arbitrary radii.

The title compound **1** crystallizes in the *P*2_1_/*n* space group with one molecule in the asymmetric unit. The molecular structure with the atom labels is shown in Figure 2 and selected bond lengths and angles are listed in Table 1. 

The bond lengths and angles are within the normal ranges [12]. The geometric parameters of the 1,2,5-thiadiazole ring in **1** are almost the same as those reported for 3,4-diphenyl-1,2,5-thiadiazole [13]. The molecular framework excluding C7 and C8 is planar, where the maximum and r.m.s. deviations of fitted atoms from the least-squares plane are 0.193(1) for S7 and 0.075 Å, respectively. Two 1,3-dithiole rings are planar (r.m.s. deviations of 0.011 for S1/S2/S3/N1/N2/C1/C2/C3 and 0.037 Å for S4/S5/C4/C5/C6 from their least-squares planes) and the angle between the least-squares planes is 3.01(8)°. These facts contrast with cases of most neutral TTF derivatives. For example, tetramethylthiotetrathiafulvalene [14] and bis(ethylenedithio)tetrathia-fulvalene [15] are bent at the central C=C bond, where the angles between the least-squares planes for two 1,3-dithiole rings are 36.2(1) and 23.9(1)°, respectively. The planarity of **1** may be ascribed to the extension of *π*-conjugated system with a 1,2,5-thiadiazole ring. One methylthio group (S7–C8) is nearly perpendicular to the molecular plane [torsion angles of 95.6(3) for C8–S7–C6–S5 and -28.2(3)° for C7–S6–C5–S4].

**Table 1:** Selected geometric parameters of **1**.

*Bond lengths (Å)*		*Bond angles (°)*	
S1–C1	1.756(3)	C2–S1–C1	93.97(15)
S2–C1	1.766(3)	C3–S2–C1	93.83(16)
S3–N2	1.643(3)	N2–S3–N1	99.39(15)
S3–N1	1.649(3)	C5–S4–C4	95.28(15)
S4–C4	1.757(3)	C6–S5–C4	95.64(14)
S5–C4	1.752(3)	C2–N1–S3	105.9(3)
N1–C2	1.318(4)	C3–N2–S3	106.2(2)
N2–C3	1.314(4)	N1–C2–C3	114.2(3)
C1–C4	1.353(4)	N2–C3–C2	114.3(3)
C2–C3	1.428(5)		

**Figure 3:**
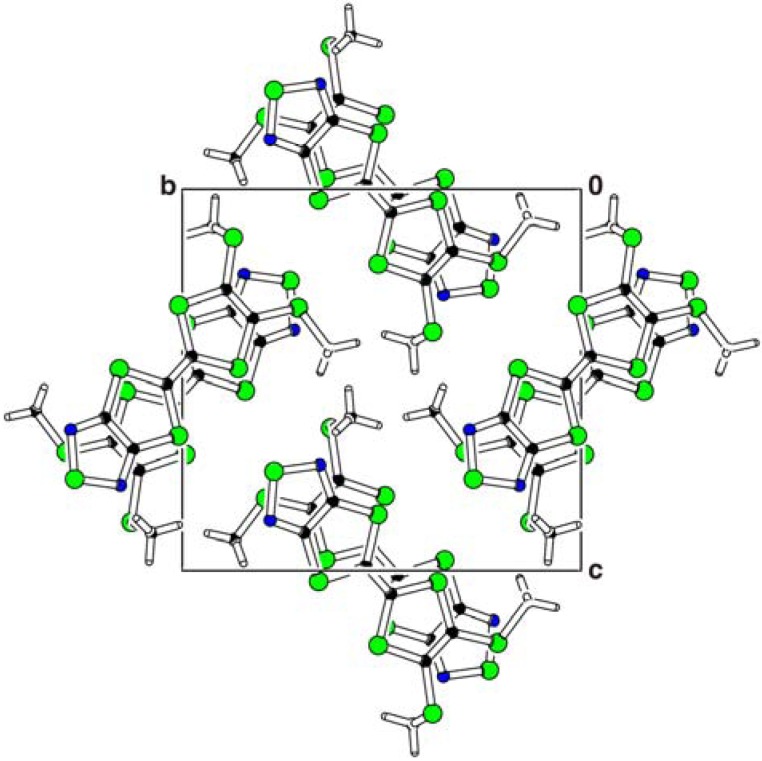
The packing diagram of **1**, viewed along the *a* axis.

**Figure 4:**
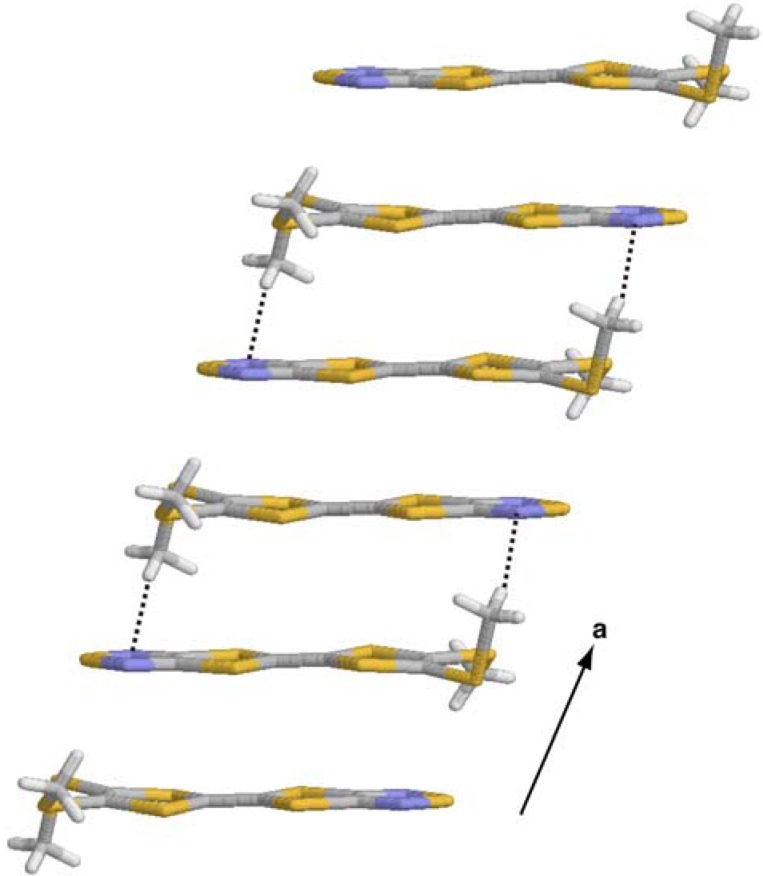
The stacking pattern of the dimer in the crystal structure of **1**. Dotted lines show intermolecular C–H···N hydrogen bonds.

The packing diagram of **1** viewed along the *a* axis is shown in Figure 3. The molecules form a head-to-tail type of *π*-stacking dimer and the dimer stacks along the *a* axis (Figure 4). 

**Figure 5:**
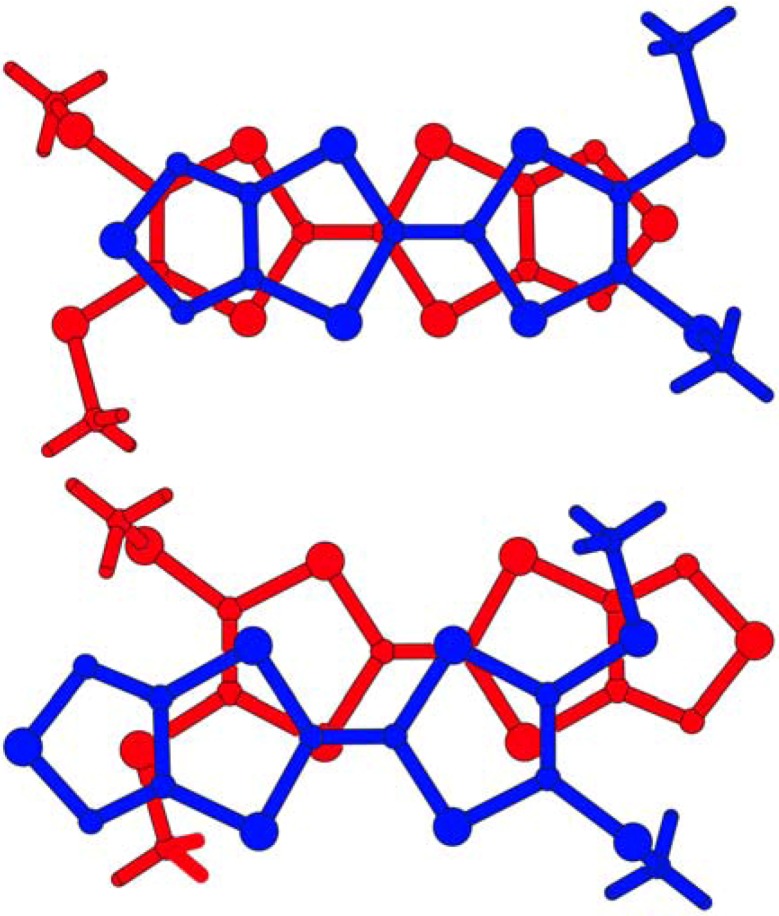
The overlap modes of the dimer in the crystal structure of **1**, the intradimer (upper) and interdimer (lower) overlapping.

In the dimer, an interplanar distance is 3.50(1) Å and the S7–C8 methylthio groups face away from each other. On the other hand, the dimers are linked by an intermolecular C8–H8A···N1(*–x*, *–y* + 1, *–z*) [3.382(5) Å] hydrogen bond and an interdimer distance is 3.71(1) Å. Between the dimers, the S7–C8 methylthio groups are placed across from each other. The overlap modes of the dimer of **1** are shown in Figure 4. In the intradimer overlapping, long axes of the molecules are fully superposed, while less overlap of *π*-conjugated system is observed in the interdimer overlapping. This is due to steric hindrance of the S7–C8 methylthio group. The intradimer arrangement of the molecules corresponds to the overlap of HOMO and LUMO for **1**. As illustrated in Figure 5, electron transfer from the large lobe of HOMO on the 1,3-dithiol-2-ylidene unit to the large lobe of LUMO on the 1,2,5-thiadiazole ring causes the intradimer overlapping in Figure 5.

The molecules **1** are also linked by weak intermolecular S···S heteroatom interactions [3.497(1) for S2···S7(*x* + 1/2, –*y* + 3/2, *z* + 1/2) and 3.572(1) Å for S5···S6(*x* + 1/2, –*y* + 3/2, *z* + 1/2)] in the crystalline state. The S···S interactions are 0.8-2.9 % shorter than the sum of the corresponding van der Waals radii and build up a unique one-dimensional molecular tape structure (Figure 7). The molecular tape is flat and takes a zigzag conformation. The crystal structure of **1** differs dramatically from that of 4,5-ethylenedioxy[1,2,5]thiadiazolotetrathiafulvalene, in where a T-shaped molecular arrangement connected with intermolecular S···N interactions was observed [6]. The conformations around the methylthio groups of **1** bring the formation of the molecular tape structure with the S···S interactions. No short intermolecular S···N interaction was found in the crystal structure of **1**.

**Figure 6:**
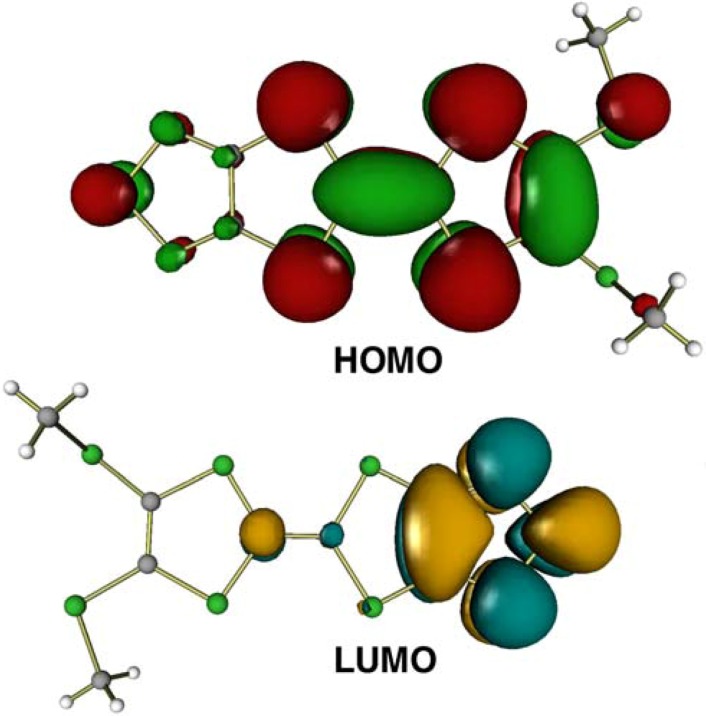
The HOMO and LUMO of **1** by single point 3-21G calculations. The energy levels of the HOMO and LUMO are calculated to be –7.55 and 1.69 eV, respectively.

**Figure 7:**
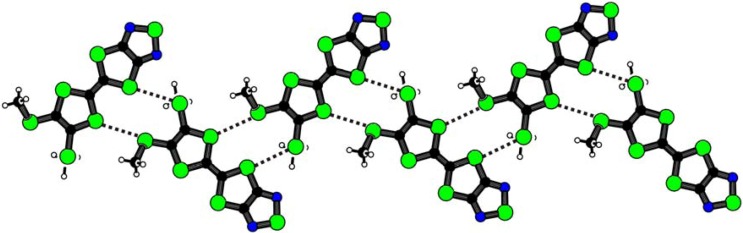
The one-dimensional molecular tape structure in the crystal structure of **1**. Dotted lines show intermolecular S···S heteroatom interactions.

## Conclusions

We have determined the crystal structure of the unsymmetrical tetrathiafulvalene derivative with a fused 1,2,5-thiadiazole ring and methylthio groups by X-ray diffraction. The molecular framework is planar within 0.19 Å. The molecules form a head-to-tail type of *π*-stacking dimer with an interplanar distance is 3.50(1) Å, where methylthio groups face away from each other. A unique one-dimensional molecular tape network can be built by the usage of intermolecular S···S heteroatom interactions [3.497(1) and 3.572(1) Å] as a connection tool.

## Experimental

### General

The title compound **1** was synthesized by the reaction of [1,2,5]thiadiazolo-1,3-dithiol-2-one with 4,5-dimethylthio-1,3-dithiole-2-thione according to the literature method [5]. Orange crystals of **1** suitable for X-ray analysis were grown from a dichloromethane solution.

### X-Ray crystallography

The data of a single crystal with a size of 0.35 × 0.05 × 0.05 mm was collected at 295 K on a Rigaku Mercury CCD diffractometer. No absorption correction was applied. The crystal structure was solved by direct methods and refined by full-matrix least-squares on *F*^2^ with SHELXS97 and SHELXL97 [16]. All non-hydrogen atoms were refined anisotropically. After anisotropic refinement of all non-hydrogen atoms, all hydrogen atoms were placed in geometrically calculated positions and refined using a riding model, with C–H = 0.96 Å and *U*_iso_(H) = 1.5*U*_eq_(C). The final least-squares cycle was based on 2,555 observed reflections [*I* > 2*σ*(*I*)] and 156 variable parameters, converged with *R*_1_ = 0.055 and *wR*_2_ = 0.125. Crystal data and refinement details are summarized in Table 2. All molecular and crystal graphics were drawn using PLATON [17] and RasMol [18]. CCDC 675501 contains the supplementary crystallographic data for this paper. These data can be obtained free of charge *via* http://www.ccdc.cam.ac.uk/data_request/cif, by e-mailing data_request@ccdc.cam.ac.uk or by contacting The Cambridge Crystallographic Data Centre, 12, Union Road, Cambridge CB2 1EZ, UK; Fax: +44-1223-336033.

**Table 2:** Crystal data and refinement details for **1**.

Chemical formula	C_8_H_6_N_2_S_7_
Formula weight	354.64
Temperature	295(1) K
Wavelength	0.71070 Å
Crystal system	Monoclinic
Space group	*P*2_1_/*n*
*a*	7.9119(8) Å
*b*	12.6713(14) Å
*c*	13.3589(15) Å
*β*	93.868(1)°
*V*	1336.2(3) Å^3^
*Z*	4
Calculated density	1.763 Mgm^-3^
Absorption coefficient	1.155 mm^-1^
*F*(000)	720
Crystal size	0.35 × 0.05 × 0.05 mm
*θ* Range for data collection	3.04–27.48°
Index ranges	–10 ≤ *h* ≤ 9
	–16 ≤ *k* ≤ 16
	–14 ≤ *l* ≤ 17
Completeness to *θ*	95.9 %
Reflections collected	12,282
Independent reflections	2,945 [ *R*_int_ = 0.0356]
Absorption correction	None
Refinement method	Full-matrix least-squares on *F*^2^
Data/restrains/parameters	2,945/0/156
Goodness-of-fit on *F*^2^	1.186
Final *R* indices [*I* > 2*σ*(*I*)]	*R*_1_ = 0.0547, *wR*_2_ = 0.1250
*R* indices (all data)	*R*_1_ = 0.0687, *wR*_2_ = 0.1330
Largest diff. peak and hole	0.483 and –0.362 eÅ^-3^
CCDC Deposition number	CCDC 675501

### Theoretical calculations

The molecular orbital shapes of HOMO and LUMO of **1** were evaluated in single point 3-21G calculations using the crystallographic geometry of **1** with Gaussian 98 [19] and were visualized with PGV [20].
